# Application of smart chemometric models for spectra resolution and determination of challenging multi-action quaternary mixture: statistical comparison with greenness assessment

**DOI:** 10.1186/s13065-024-01148-9

**Published:** 2024-03-02

**Authors:** Aya A. Mouhamed, Ahmed H. Nadim, Nadia M. Mostafa, Basma M. Eltanany

**Affiliations:** Department of Pharmaceutical Analytical Chemistry, Faculty of Pharmacy, Cairo University, Cairo, 11562 Egypt

**Keywords:** Multivariate models, Greenness assessment, Paracetamol, Chlorpheniramine maleate, Caffeine, Ascorbic acid

## Abstract

A multivariate spectrophotometric method is a potential approach that enables discrimination of spectra of components in complex matrices (e.g., pharmaceutical formulation) serving as a substitution method for chromatography. Four green smart multivariate spectrophotometric models were proposed and validated, including principal component regression (PCR), partial least-squares (PLS), multivariate curve resolution-alternating least squares (MCR-ALS), and artificial neural networks (ANN). The developed chemometric models were compared to resolve highly overlapping spectra of Paracetamol (PARA), Chlorpheniramine maleate (CPM), Caffeine (CAF), and Ascorbic acid (ASC). The four multivariate calibration models were assessed via recoveries percent, and root mean square error of prediction. Hence, the proposed models were efficiently applied with no need for any preliminary separation step. The models were utilized to analyze the studied components in their combined pharmaceutical formulation (Grippostad® C capsules). Analytical GREEnness Metric Approach (AGREE) and eco-scale tools were applied to assess the greenness of the established models and found to be 0.77 and 85, respectively. Moreover, the proposed models have been compared to official ones showing no considerable variations in accuracy and precision. Therefore, these models can be highly advantageous for conducting standard pharmaceutical analysis of the substances researched within product testing laboratories.

**Supplementary Information:**

The online version contains supplementary material available at 10.1186/s13065-024-01148-9.

## Introduction

**Fig. 1:**
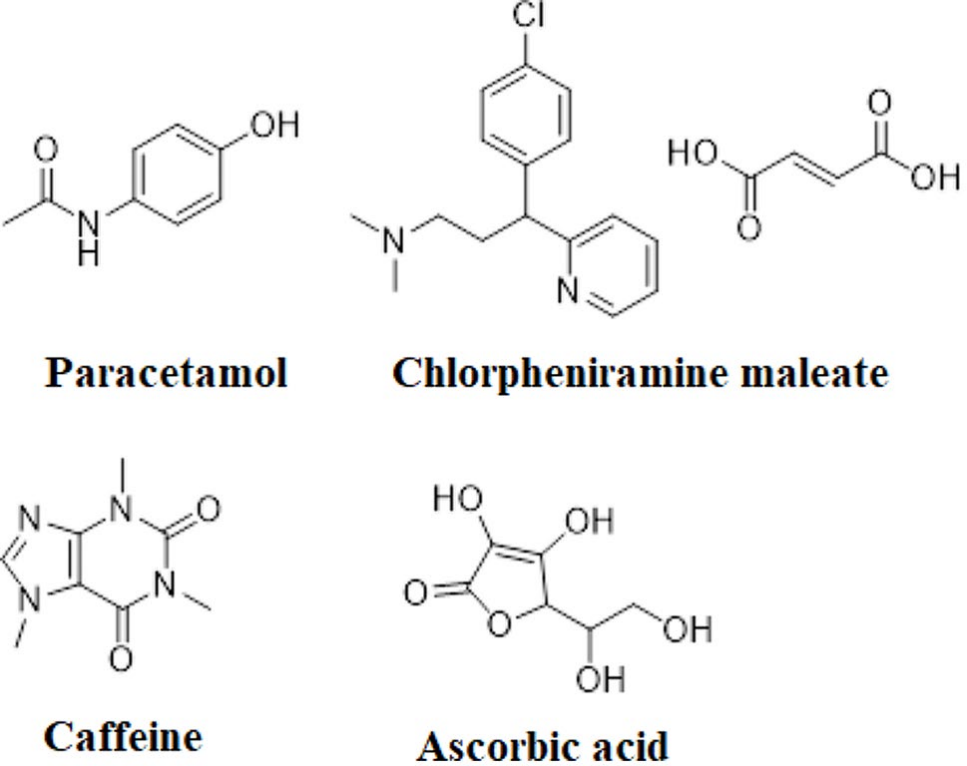
Chemical structure of the studied drugs

Quality control analysis within the pharmaceutical industry requires determining several parameters for both raw materials and end products. The most utilized analytical technique for quality control analysis of pharmaceutical products is high-pressure liquid chromatography (HPLC) [1]. However, an HPLC technique is costly, requires significant labor, and consumes time, whilst also generating hazardous waste materials. This gets an interest in developing simple, green, and valid alternative techniques that produce precise and accurate outcomes with efficiency and minimal human intervention [2]. Chemometrics is a well-known chemical discipline that uses mathematics, statistics, and formal logic for extracting meaningful and important qualitative or quantitative information from given chemical data [3]. Recently, chemometric models like principal component regression (PCR), partial least squares (PLS), multivariate curve resolution alternating least squares (MCR-ALS), and artificial neural networks (ANN) have generated considerable interest in the detection of multi-component preparations [4–8]. PCR and PLS are the most applied multivariate calibration approaches in chemometrics. These models enable the resolution of overlapped spectra, reduction of interference between signals, and minimization of noise [9]. Newer advanced models were implemented for multivariate calibration in recent years, one of which is MCR-ALS. ALS was then applied, and several constraints were tried to limit the possible solutions and improve the quantification of compounds’ concentration profiles [10, 11]. ANNs represent a sophisticated model capable of emulating several cognitive processes of the human brain through diverse algorithms. It is deemed superior to other conventional multivariate models to model variables’ linear and nonlinear relationships [11, 12]. Green chemistry has become the main driving force in both laboratory and industry settings in promoting sustainability. The twelve principles of Green Analytical Chemistry (GAC) developed by Anatas provide guidance for those interested in pursuing this approach [13]. Chemists from various fields, including organic, analytical chemistry, and chemical engineering provide a framework for implementing measures to increase the eco-friendliness of chemical materials and processes [14]. The bulk of endeavors to create more environmentally friendly chemical processes concentrate on employing cleaner, less hazardous, gentler solvents, or removing solvents altogether, also minimizing chemical reagents. Other efforts involve preserving energy through the use of underivatized samples and employing raw materials derived from renewable resources [15]. Paracetamol (PARA), N-acetyl-p-aminophenol, or acetaminophen is commonly used as an antipyretic and analgesic drug. It is a painkiller that can alleviate symptoms of cold including headache, earache, and joint pain. Additionally, it is effective in lowering a high body temperature [16, 17] **(**Fig. 1**)**. Chlorpheniramine maleate (CPM), (3*RS*)-3-(4-Chlorophenyl)-*N*, *N*-dimethyl-3-(pyridine-2-yl) propane-1-amine hydrogen (*Z*)- butenedioate, is an antihistaminic drug that is used to treat runny nose, sneezing, and watery eyes caused by the common cold, or the flu [16, 17] **(**Fig. 1**)**. Caffeine (CAF), 1,3,7-Trimethyl-3,7-dihydro-1 H-purine-2,6-dione, is a CNS stimulant that improves alertness and alleviates the malaise that is often associated with the common cold [16, 17] **(**Fig. 1**)**. Ascorbic acid (ASC) is an antioxidant substance that supports the immunity of the body and helps fight against cold [16]. It possesses a positive impact and plays a protective role in curing new coronavirus disease [18]. It is chemically designated as (5*R*)-5-[(1*S*)-1,2-Dihydroxyethyl]-3,4-dihydroxyfuran-2(5*H*)-one [17] **(**Fig. 1**)**. A combination of PARA with CPM, CAF, and ASC is used to treat common symptoms like headaches, limb pain, rhinitis, and dry cough that occur with the common cold [19]. It is also an effective treatment for the same symptoms that are associated with COVID-19 [20]. Although different chemometric assays were reported for the determination of the investigated drugs either in different binary [21–23]or in a quaternary mixture [24] a comprehensive literature review uncovered no evaluated chemometric models for resolving the spectra of the four drugs in their dosage form. In addition, these reported techniques did not consider the greenness assessment. The present eco-friendly work seeks to reduce the use of potentially dangerous materials that harm the environment. Hither, AGREE and penalty points scoring systems were employed to evaluate the greenness of our developed models. AGREE provides a thorough environmental assessment of the whole analysis procedure [25]. The eco-scale assessment of the established approaches was calculated and deducted from 100 based on penalty points [26]. This research aimed to apply green smart multivariate models to concurrently quantify PARA, CPM, CAF, and ASC in their dosage form and to quantitatively assess the efficiency of the established models and compare their performance by different statistical tests. The study was based on UV-Vis spectrophotometry as an analytical technique in combination with a non-linear model (ANN/RBF) and multivariate curve resolution.

## Experimental

### Apparatus and software

All spectrophotometric measurements were done using a Shimadzu 1605 UV- spectrophotometer (Kyoto, Japan), with 1.00 cm quartz cells at a range of (200–400 nm). MATLAB® 8.3.0.532 (R2014a), PLS Toolbox (version 2.1), MCR-ALS Toolbox (free software available at http://www.mcrals.info), and the Neural Network Toolbox™ employed in MATLAB® were used in data analysis.

### Chemicals and materials

PARA, CPM, CAF, and ASC powders were kindly supplied by the Egyptian Drug Authority, (EDA), Egypt. Their purity was tested by the official British Pharmacopeial method [17] for PARA and ASC and found to be 100.04 ± 1.26 and 100.04 ± 1.36, respectively, while by the USP official method [27]for CPM and CAF and found to be 100.00 ± 1.19 and 99.69 ± 1.73. Grippostad® C capsules (batch no. G52165) were manufactured by STADA, Germany claimed to contain 200.00 mg PARA, 2.50 mg CPM, 25.00 mg CAF, and 150.00 mg ASC per capsule. Methanol was purchased from Sigma-Aldrich, Germany.

### Standard solution

Stock standard solutions of PARA, CPM, CAF, and ASC (1.00 mg/mL, each) were prepared by weighing 100.00 mg of each drug into four separate 100 mL-volumetric flasks, then methanol was added. The solutions were sonicated until dissolution and then completed to the mark with methanol. Working standard solutions with a concentration of 100.00 µg mL^− 1^ from PARA, CPM, CAF, and ASC were prepared from their corresponding stock standard solutions.

### Procedure

#### Spectral characteristics and absorption spectra

The absorption spectra of PARA, CPM, CAF, and ASC were measured over the range of 200.0–400.0 nm. The spectrum data points ranging from 220.0 to 300.0 nm, were selected and transferred for further data analysis on MATLAB®.

#### Construction of calibration and validation sets

A five-level, four-factor calibration design [28] was employed to construct the calibration and validation sets. Twenty-five mixtures containing various concentrations of PARA, CPM, CAF, and ASC in the ranges between 4.00 and 20.00, 1.00–9.00, 2.50–7.50, and 3.00–15.00 µg mL^− 1^, respectively were applied to design the calibration set. In 10 mL volumetric flasks, different aliquots of their working solutions were combined and diluted with methanol to the appropriate level. The spectra of the resulting solutions were measured within the wavelength range of 200.0-400.0 nm. Using 1 nm intervals, the spectral data in the 220.0–300.0 nm range were imported into MATLAB for data manipulation and calibration model building. In the calculations, 81 experimental points were utilized. The spectral data were mean-centered prior to the ANN, PLS, PCR, and MCR-ALS model construction. For both the PCR and PLS models, latent variable (LV) numbers were optimized using leave-one-out cross-validation. Four LVs that corresponded to the least significant error of calibration were optimum in both models. In the MCR-ALS model, non-negativity constraints were chosen which oblige concentration to be zero or more than zero. In this study, we established a feed-forward model based on Levenberg–Marquardt backpropagation as an ANN training algorithm [8]. To achieve optimal network architecture, various elements require refinement: the number of nodes in the hidden layer, the learning rate, and the number of epochs. Four hidden neurons were found to be optimum when using a purelin-purelin transfer function. Further parameters like a learning rate of 0.1 and 100 epochs were optimized as well. The calibration models of PCR, PLS, MCR-ALS, and ANN were constructed, and their predictive power was evaluated using a validation set consisting of five samples.

#### Assay of pharmaceutical dosage form

The contents of ten Grippostab®capsules were accurately emptied and weighed. The equivalent weight to one capsule was added into a 100-mL volumetric flask, 25 mL methanol was added then the solution was ultrasonicated for 30 min then filtered into a 100-mL measuring flask and the volume made up to the mark with methanol. 0.50 mL from the previous solution was transferred into a 50-mL volumetric flask and then completed volume with methanol after spiking with 0.75 µg mL^− 1^ of standard working solution of CPM. The preceding solution was claimed to have a final concentration of 20.00 µg mL^− 1^ PARA, 1.00 µg mL^− 1^ CPM, 2.50 µg mL^− 1^ CAF, and 15.00 µg mL^− 1^ ASC.

The aforementioned procedures were utilized to examine pharmaceutical preparation via the proposed chemometric models. Subsequently, the concentrations of PARA, CPM, CAF, and ASC were determined.

## Results and discussion

**Fig. 2:**
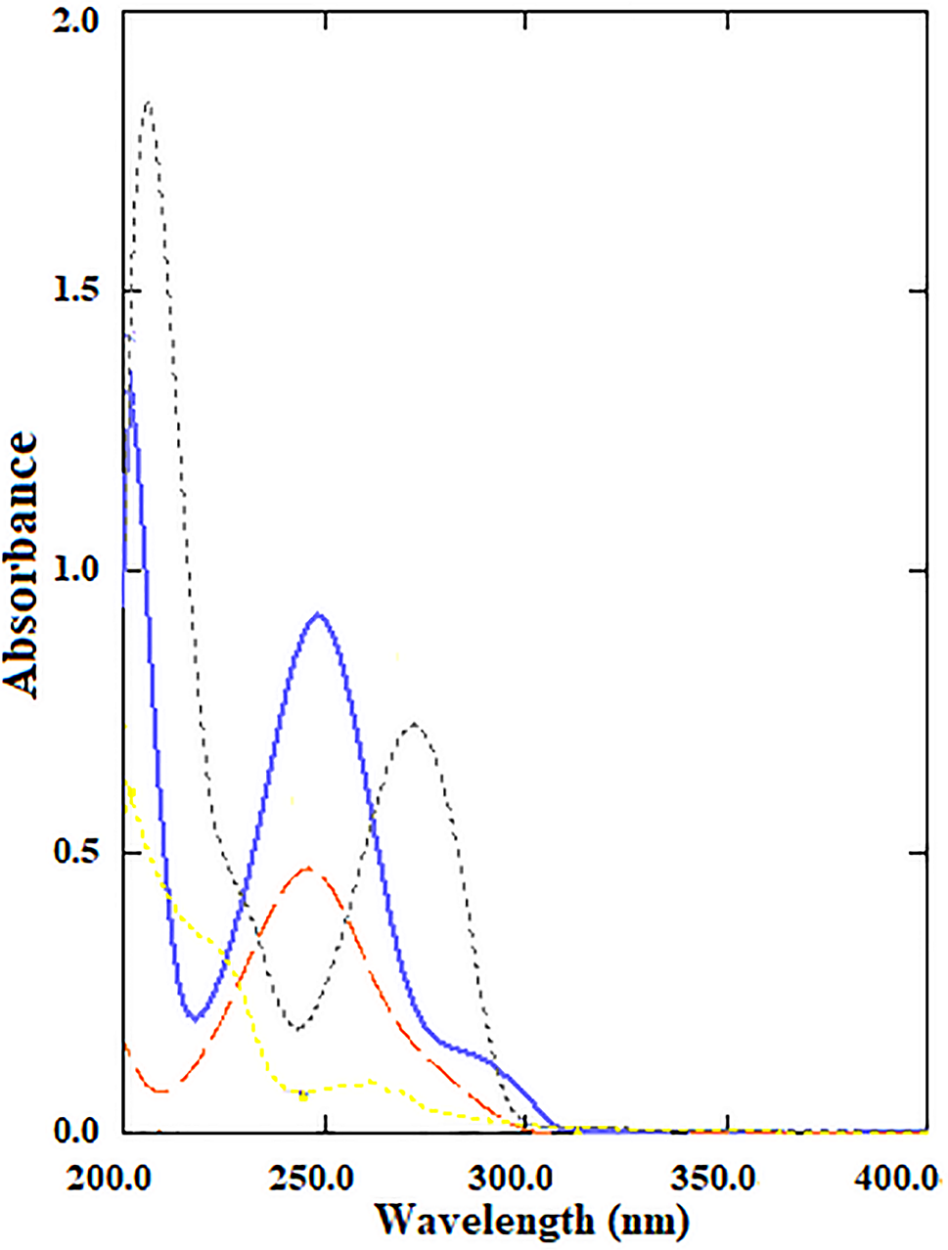
Absorption spectra of 12.00 µg mL^− 1^ PARA (—), 1.00 µg mL^− 1^ CPM (….), 2.50 µg mL^− 1^ CAF(…), and 6.00 µg mL^− 1^ ASC (— —), using methanol as solvent

The use of multivariate analysis as a tool to resolve severely overlapping spectroscopic data simultaneously including numerous spectroscopic variables at different wavelengths instead of using univariate analysis that relies on a single value corresponding to a maximum absorbance at selected wavelength leads to an increase in specificity and sensitivity [29]. Moreover, multivariate calibrations are efficiently used in biodiesel, plant extract, and pharmaceutical formulation analysis [30–33]. Herin, the quantification of our combined drugs in Grippostad C® capsules using the univariate spectrophotometric method was hindered by the severe spectral overlap **(**Fig. 2**)**. Hence, chemometric models (PCR, PLS, MCR-ALS, and ANN) were employed to quantify them successively.

### Calibration and validation set

The suggested models were optimized and calibrated using twenty-five mixtures, Table 1. The samples’ absorbance data was scanned between 220.0 and 300.0 nm. This range was chosen since all components have suggested absorbance characteristics within this range. To remove noise influence within the calibration matrix., wavelengths below 220.0 nm were excluded. Wavelengths over 300.0 nm were also excluded because they were regarded as less informative absorbance data.

**Table 1:** **Concentrations of PARA, ASC, CAF, and CPM in the calibration and validation sets for the multivariate calibration models**

Mix no.	Conc. (µg mL^− 1^)			
	PARA	ASC	CAF	CPM
1	12.00	9.00	7.50	5.00
2	12.00	6.00	5.00	9.00
3	8.00	3.00	5.00	9.00
4	4.00	6.00	12.50	9.00
5	8.00	15.00	12.50	5.00
6	20.00	15.00	7.50	1.00
7	20.00	9.00	2.50	9.00
8	12.00	3.00	12.50	1.00
9	4.00	15.00	2.50	7.00
10	20.00	3.00	10.00	7.00
11	4.00	15.00	12.50	5.00
12	16.00	12.00	7.500	9.00
13	12.00	15.00	10.00	9.00
14	20.00	12.00	12.50	3.00
15	16.00	15.00	5.00	3.00
16	20.00	6.00	5.00	5.00
17	8.00	6.00	7.50	7.00
18	8.00	9.00	10.00	3.00
19	12.00	12.00	5.00	7.00
20	16.00	6.00	10.00	1.00
21	8.00	12.00	2.50	1.00
22	16.00	3.00	2.50	5.00
23	4.00	3.00	7.50	1.00
24	4.00	9.00	5.00	1.00
25	12.00	9.00	7.50	5.00
**26**	**20.00**	**15.00**	**2.50**	**1.00**
**27**	**4.00**	**15.00**	**2.50**	**9.00**
**28**	**8.00**	**8.00**	**8.00**	**8.00**
**29**	**12.00**	**9.00**	**7.50**	**5.00**
**30**	**20.00**	**3.00**	**12.50**	**1.00**

^*^The bold numbers represent the validation set.

### PCR and PLS models

**Fig. 3:**
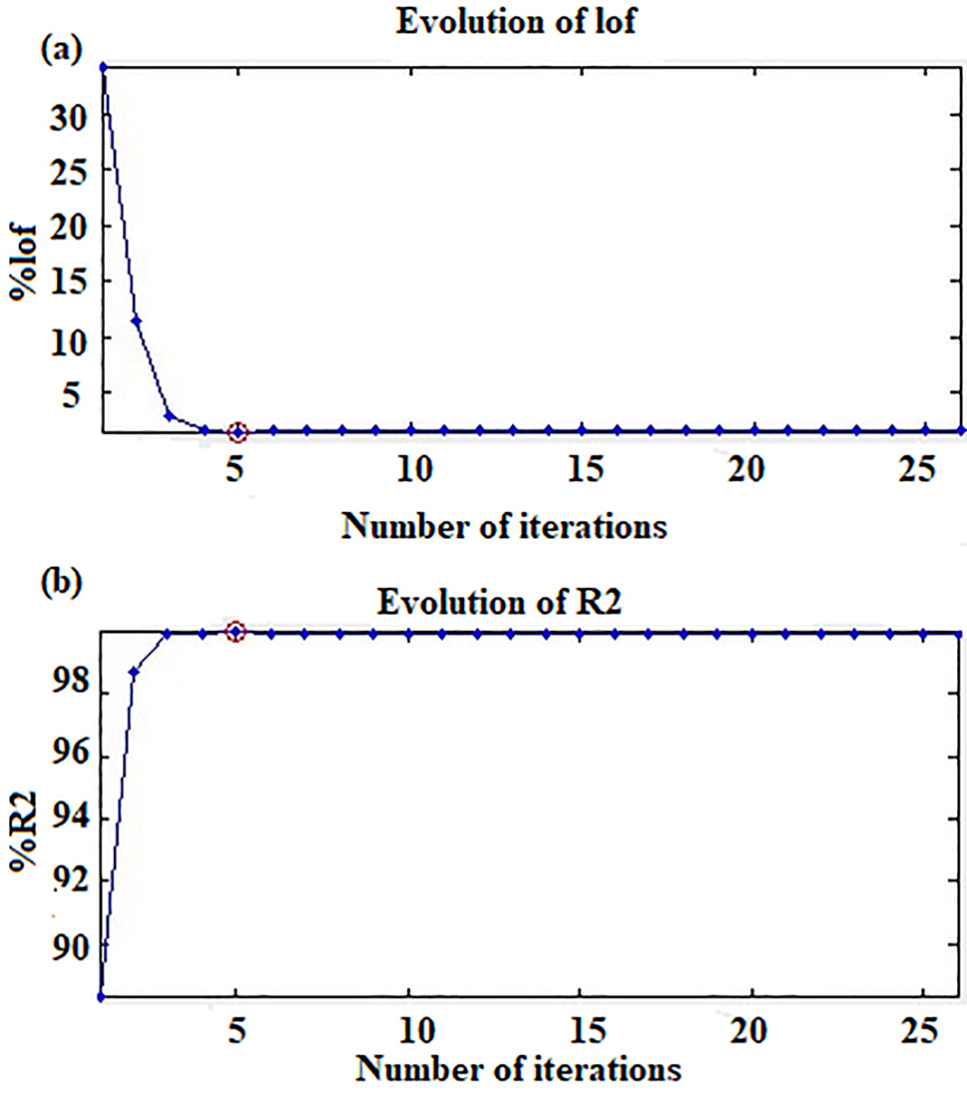
RMSEC plot of the cross-validation results of the calibration set as a function of the number of latent variables used to construct (a) PLS calibration and (b) PCR calibration

PCR and PLS have attracted significant attention in chemometrics for multicomponent analysis. Determining which method is superior remains a challenging problem [34–36]. These models are particularly effective when there is only limited information available regarding the components. PCR generates components to increase the interpretability of data, without taking the response variable into consideration. Conversely, PLS involves the response variable in its analysis and frequently produces models that require fewer components to fit the response variable [36]. Hence, PLS produces a more resilient model by eliminating interference from absorbance and concentration data. To establish the optimal number of variables, a cross-validation approach of leaving out one sample at a time was applied **(**Fig. 3**)**. In our investigation, four LVs proved to be optimal in both PCR and PLS.

### MCR-ALS model

**Fig. 4:**
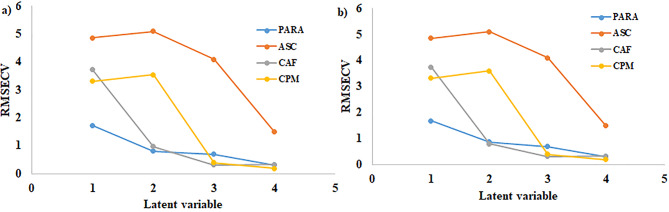
(a) Percentage lack of fit and (b) variance percentage of MCR-ALS model

MCR constructs a regression model by evaluating the relationship of a variable with other variables [30, 37]. This model extends the Beer-Lambert law into the multi-wavelength domain [38]. MCR separates the spectral data background into concentration profile and pure spectral-profile matrices. Then the matrix of residuals was calculated. MCR-ALS iteratively estimates the concentrations of proposed components from spectral profiles. Numerous constraints such as unimodality, closure, equality, and non-negativity were applied during ALS optimization to model concentration and pure spectra profile shape. Another advanced constraint was applied in MCR-ALS framework to obtain pure resolved profiles in arbitrary units without reference quantitative information [39]. This issue has been resolved by incorporating an inner calibration into the MCR-ALS model through correlation constraint [39]. During ALS optimization, the order of components could be permuted without changing the data matrix because of rotational ambiguity [37]. In this study, non-negativity constraints with correlation constrain were applied to both the concentration and spectral profiles. Convergence was commonly reached when equal to a value of (0.1%) [40]. The convergence was achieved after five iterations. The figures of merit of optimization procedures (% lack of fit and variance percentages (R^2^)) were calculated and found to be 1.5625 and 99.9754 which were reasonable and assisted in improving the strength of the developed MCR-ALS model **(**Fig. 4**)**.

**Fig. 5:**
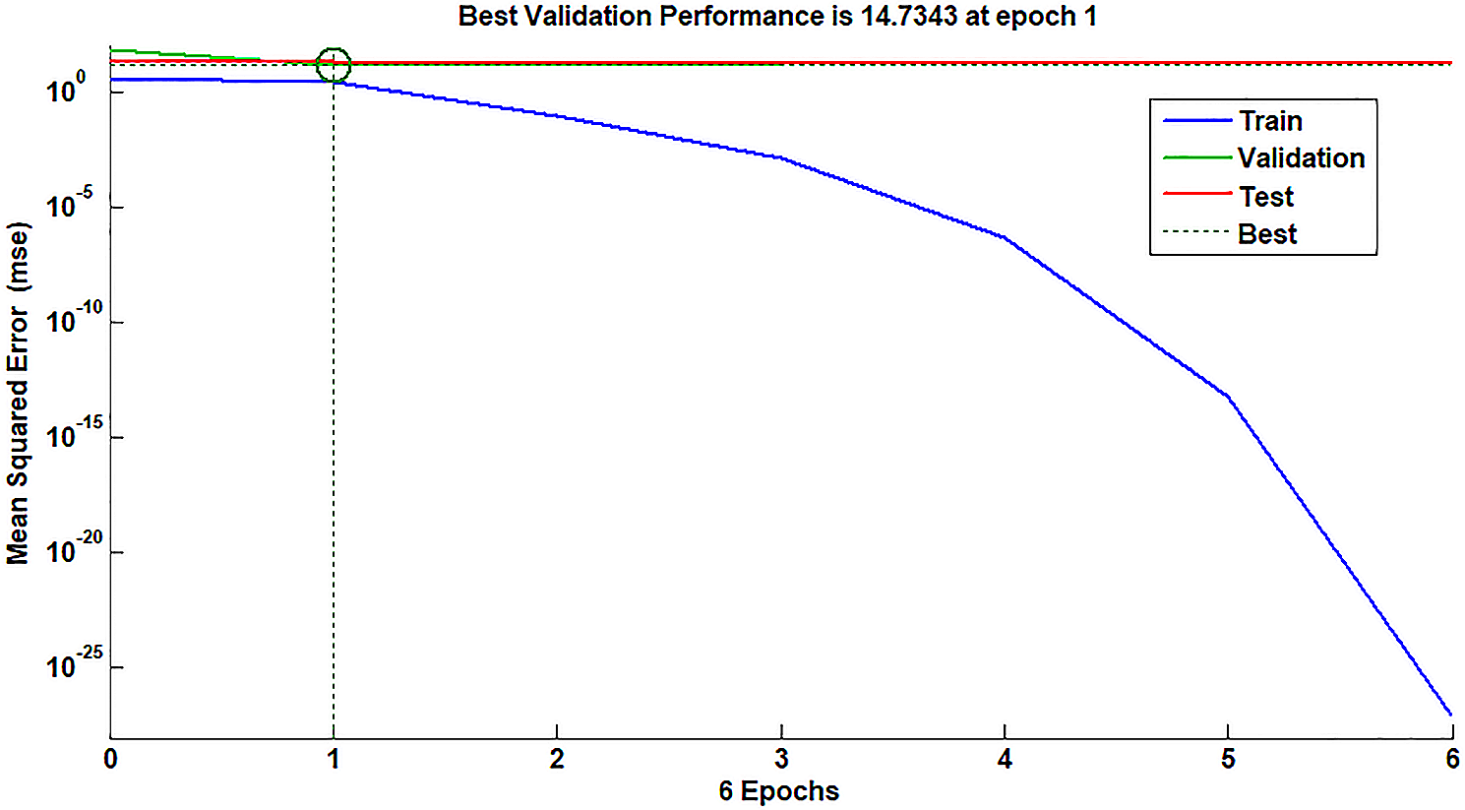
Pure spectra (—) and MCR-ALS (….) for (a) PARA(b) CPM (c) CAF (d) ASC.

The MCR-ALS model can estimate the spectral profiles of drugs, providing a qualitative meaning in its algorithms. The estimated spectra were closely similar to the original ones **(**Fig. 5**)**. To calculate the concentration of the studied components in the validation set, the related spectral and concentration profiles were recovered during the ALS optimization using a one-by-one test sample as recommended in multivariate calibration models [41]. The predicted concentrations are presented in (**Table ****S1**) with a good value of RMSEP. The MCR-ALS model can estimate the spectral profiles of drugs, providing a qualitative meaning in its algorithms. The estimated spectra were closely similar to the original ones **(**Fig. 5**)**. Therefore, the MCR-ALS model offers the added benefit of qualitative component detection, in addition to its quantitative determination ability.

### ANN model

**Fig. 6:**
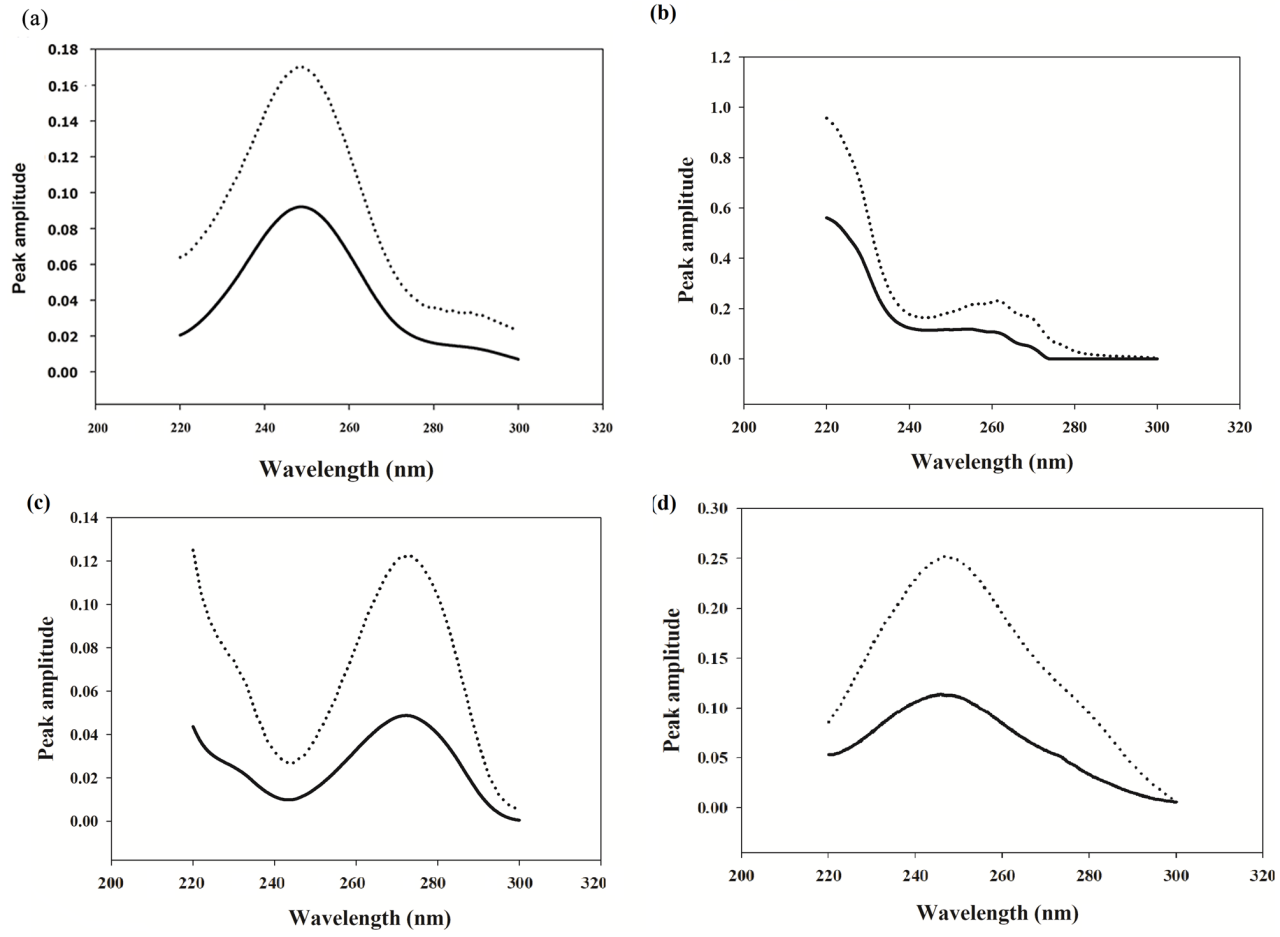
Best validation performance for the prediction of the ANN model

**Fig. 7:**
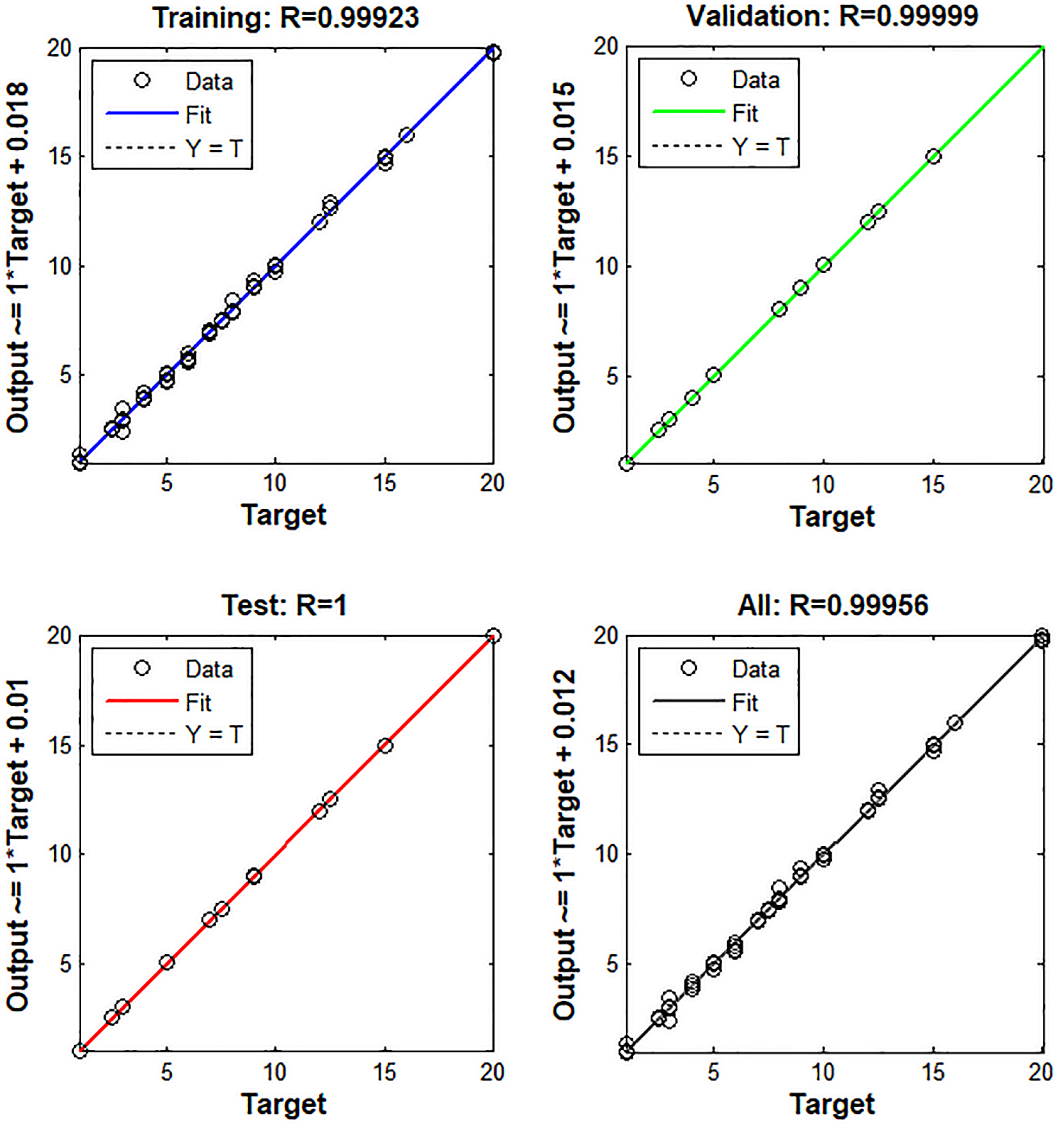
Prediction for the training, test, and validation diagrams of the ANN model

It is a rivaled intelligence technique comprised of a significant number of simple, meticulously connected nodes or synthetic neurons that mimic the authentic nervous system function to identify correlations between inputs and outputs. ANN is a more effective option for modelling both linear and non-linear relationships between variables than other established multivariate approaches, such as PCR and PLS [11, 42]. The ANN type that was trained in this research is the feed-forward model. 81 neurons were used as an input layer, corresponding to the number of spectral data points used, and for the output layer, four neurons were used which corresponded to the number of compounds to be established in each sample. The optimum number of neurons in the hidden layer was five using a purelin-purelin transfer function and 100 epochs. A fully trained ANN’s mean squared error (MSE) performance over epochs is shown in Fig. 6. The MSE of training was decreased steadily after epoch = 0. Both the test and validation plots exhibited a similar pattern without abrupt variation. Figure 7 also shows the predictions for training, test, and validating sets diagrams of the chosen layers and neurons.

To evaluate the predictive ability of the chemometric models PCR, PLS, MCR-ALS, and ANN models, spectra from the validation set were utilized. The average recoveries and RSDs were computed for each component **(**Table 2**)**, indicating favorable outcomes. Table 3 offers the regression and validation parameters for the validation sets to quantify pure samples of PARA, CPM, CAF, and ASC. To judge the performance of the proposed multivariate calibration models, three statistical tests were calculated. The first statistical test was Durbin-Watson statistical test which predicted the correlation among prediction residuals [43–45]. The second statistical test was the root mean square error of prediction (RMSEP) [46]. RMSEP is mostly recognized as a measuring tool for the evaluation of prediction quality [46]. The estimation of RMSEP plays a key role in the validation of multivariate calibration models as it indicates both the accuracy and precision of the model [46]. The third statistical test was the elliptical joint confidence region (EJCR) test [47, 48]. EJCR test was conducted to compare the performance of ANN and MCR-ALS. Durbin-Watson indicator showed a very low associated probability in each of the four individual analytes in Linear models (PCR, PLS) **(**Table 3**)**, indicating that non-linearity was significant. Moreover, the non-linear ANN model had the least RMSEP and RMSEC **(**Table 3**)**. The performance of ANN when compared with MCR-ALS was statistically demonstrated using an EJCR test, showing that there was no statistical difference between the two models **Figure ****S1**. All results confirmed the idea that ANN is the model of choice for the quantitative determination of the studied drug mixture. Additionally, only the MCR-ALS model can separate the pure spectral profiles of the four components. As a result, it was suitable for both qualitative and quantitative analysis.

**Table 2:** Prediction of validation set samples using the proposed chemometric models

Sample No.	Concentration (µg mL^− 1^)				PCR				PLS			
					%Recovery				%Recovery			
	PARA	ASC	CAF	CPM	PARA	ASC	CAF	CPM	PARA	ASC	CAF	CPM
26	20.00	15.00	2.50	1.00	98.88	98.96	101.91	100.96	98.88	96.93	101.87	101.07
27	4.00	15.00	2.50	9.00	102.54	98.72	98.96	98.37	102.44	98.39	98.92	98.42
28	8.00	8.00	8.00	8.00	99.91	98.83	99.53	100.48	100.01	99.25	99.54	100.53
29	12.00	9.00	7.50	5.00	99.40	101.87	96.16	99.87	99.44	101.47	96.16	99.92
30	20.00	3.00	12.50	1.00	99.81	100.61	96.06	100.08	99.86	101.49	96.07	100.51
**Mean**					100.11	99.80	98.53	99.59	100.12	99.51	98.51	100.09
**SD**					**1.42**	1.39	2.47	0.749	1.36	1.99	2.45	1.02
					**MCR-ALS**				**ANN**			
26	20.00	15.00	2.50	1.00	100.87	101.02	98.95	102.64	99.83	100.00	100.01	100.00
27	4.00	15.00	2.50	9.00	102.62	98.70	96.49	96.51	100.00	100.00	100.00	100.00
28	8.00	8.00	8.00	8.00	98.08	101.97	98.02	99.35	100.00	100.00	100.00	104.52
29	12.00	9.00	7.50	5.00	99.74	101.57	96.13	100.10	101.16	99.52	101.95	100.00
30	20.00	3.00	12.50	1.00	100.48	101.57	99.43	100.64	100.00	100.00	100.00	100.00
**Mean**					100.29	99.88	97.80	99.85	100.20	99.88	100.49	100.63
**SD**					0.58	0.24	1.46	2.23	0.54	0.24	0.98	1.26

**Table 3:** Performance parameters of the calibration and validation sets calculated for each proposed model

Parameter	PCR					PLS			
	PARA	ASC		CAF	CPM	PARA	ASC	CAF	CPM
**Slope**	0.9985	0.9822		0.9535	0.9906	0.9984	0.9631	0.9538	0.9908
**Intercept**	-0.0827	0.1268		0.1474	0.0243	-0.0755	0.2519	0.1457	0.0267
**Correlation coefficient (r)**	0.9998	0.9997		0.9994	0.9998	0.9999	0.9992	0.9994	0.9998
**RMSEC**	0.2435	0.2644		0.2532	0.1462	0.2422	0.3430	0.2532	0.1468
**RMSEP**	0.1178	0.1463		0.2255	0.0724	0.1171	0.2373	0.2247	0.0720
**Durbin-Watson DW**	0.907	1.358		0.320	1.572	1.030	0.954	0.320	1.644
**Durbin-Watson p**	˂0.0001	˂0.0001		˂0.0001	˂0.0001	˂0.0001	˂0.0001	˂0.0001	˂0.0001
**Parameter**	**MCR-ALS**					**ANN**			
	**PARA**	**ASC**	**CAF**		**CPM**	**PARA**	**ASC**	**CAF**	**CPM**
**Slope**	1.0147	0.9942	0.9939		0.9717	1.001	0.9999	0.9996	0.9976
**Intercept**	-0.1788	0.0000	0.0864		0.0703	0.024	-0.0094	0.0394	0.0456
**Correlation coefficient (r)**	0.9999	0.9998	0.9997		0.9996	0.9999	1.0000	0.9998	0.9998
**RMSEC**	0.1412	0.2156	0.1254		0.2315	0.1330	0.2102	0.1195	0.0983
**RMSEP**	0.3304	0.6973	0.6285		0.6136	0.0699	0.0714	0.0043	0.1131
**Durbin-Watson DW**	-	-	-		-	2.000	2.000	2.000	2.000
**Durbin-Watson p**	-	-	-		-	0.161	1.000	1.000	0.277

### Assay of pharmaceutical dosage form

The developed chemometric models were used for the assessment of PARA, CPM, CAF, and ASC in their dosage form. Grippostad C® Capsules contain PARA, CPM, CAF, and ASC with a challenging ratio (80:1:10:60) which permits the determination of PARA, CAF, and ASC without any interference with CPM. While CPM was quantified after spiking with 0.75 µg mL^− 1^ of standard working solution of CPM. The good recovery % data results with a standard deviation of less than 2 **(**Table 4**)**, confirm the precise quantification of the combined four drugs in their pharmaceutical product.

**Table 4:** Quantitative determination of PARA, ASC, CAF, and CPM in the dosage form by the proposed chemometric models

	Drugs	PCR	PLS	MCR-ALS	ANN
		%Recovery ± S.D.^*^			
Grippostad C® capsules	PARA	98.21 ± 0.56	98.63 ± 0.25	98.22 ± 0.58	99.12 ± 0.23
	ASC	98.56 ± 0.88	98.23 ± 0.36	99.56 ± 0.16	98.63 ± 0.45
	CAF	99.25 ± 0.69	100.22 ± 0.14	100.12 ± 0.23	99.58 ± 0.69
	CPM	97.98 ± 0.89	98.21 ± 0.26	98.78 ± 0.24	98.67 ± 0.36

^*^Average of three determination

### Greenness assessment

**Fig. 8:**
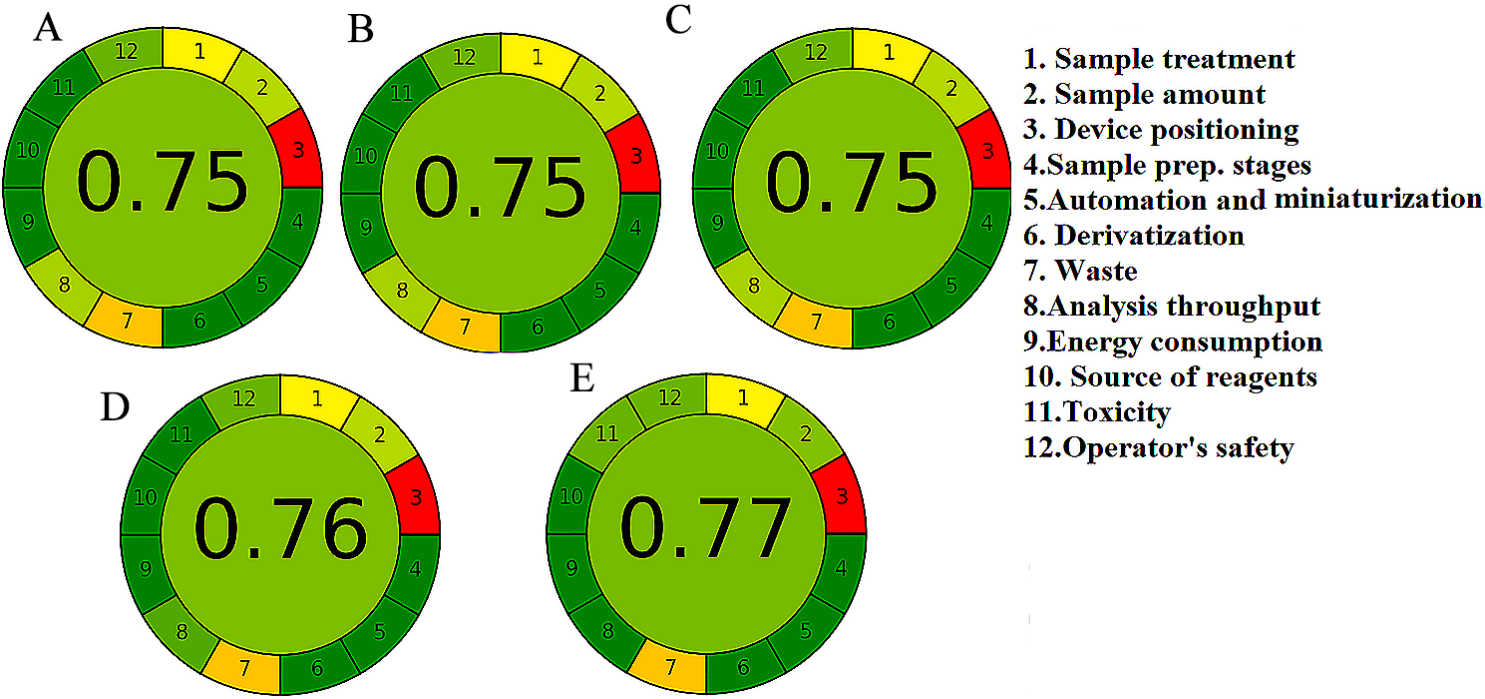
AGREE green profile assessment of the reported methods (A) [21], (B) [22], (C) [23], (D) [24], and (E) the developed chemometric methods for determination of PARA, CPM, CAF, and CPM.

To qualify analytical techniques as environmentally sustainable, it is essential to refine analytical procedures by eliminating or reducing hazardous reagents, preserving energy, and enhancing analyst safety. Refining is required throughout the process of analyzing, from gathering samples to analytical waste management [14, 49]. Therefore, it is vital to appraise the environmental impact and possible repercussions on the workforce when measuring the eco-friendliness of analytical techniques. Various assessment methods have been conceived to gauge the greenness of analytical procedures [15]. Two metrics were used to assess the greenness of the suggested technique, the Analytical Eco-Scale, and Analytical Greenness Metric, which are significant in assessing eco-friendliness. Analytical Eco-scale score was determined by subtracting the total number of penalty points for the whole procedure from a base of 100. An excellent green analysis will show a score higher than 75 [26]. The developed technique showed a high score on the Eco-scale (85) proving that it is an excellent green method of analysis **(**Table 5**)**. However, Analytical Eco-scale did not supply comprehensive information about the assessed parameters. To provide more information, the most recent greenness assessment tool, AGREE was implemented [25]. The pictogram of the proposed models scored 0.77 indicating that the method is green, Fig. 8. This came in agreement with the previously reported literature that the closer the score to one, the better the method [25]. The proposed models showed an overall excellent greenness profile. Furthermore, The environmental impact assessment of the proposed models was compared with the reported literature [21–24]as shown in Table 5**and** Fig. 8.

**Table 5:** A comparison between the developed chemometric models and the reported methods using analytical Eco-scale

Parameters	proposed models	Reported methods			
		[21]	[22]	[23]	[24]
Reagents					
Methanol Distilled water Ethanol	12 - -	- 0 -	12 - -	- 0 -	- - 8
**Instrument**					
Energy (˂ 0.1 kWh per sample	0	0	0	0	0
Occupational hazard	0	0	0	0	0
waste	3	3	3	3	3
**Total PPS**	15	3	15	3	11
**Analytical Eco-scale score**	**85 (Excellent green analysis)**	**97 (Excellent green analysis)**	**85 (Excellent green analysis)**	**(Excellent green analysis)**	**89(Excellent green analysis)**

### Statistical Analysis

The statistical analysis of the chemometric approaches developed in this study and the official methods [17, 27], presented in Table 6, demonstrated that there was no significant disparity between the two in terms of accuracy and precision.

**Table 6:** Statistical comparison of the results obtained by the proposed chemometric models and the official methods for the determination of PARA, ASC, CAF, and CPM in their pure powdered form

Paracetamol ^[a]^					
Methods	PCR	PLS	MCR-ALS	ANN	Official methods^*[a][b][c]^
Mean	100.11	100.12	100.36	100.29	101.48
S.D.	1.42	1.67	1.66	0.58	0.88
Variance	2.02	1.85	2.74	0.34	0.79
n	5	5	5	5	5
Student’s *t*-test (2.036)	1.827	2.008	1.332	2.49	
*F* value (6.39)	2.56	2.34	3.47	2.32	
**Ascorbic acid** ^**[a]**^					
Mean	99.80	99.51	100.03	99.60	98.46
S.D.	1.39	1.99	1.67	0.79	0.92
Variance	1.93	3.98	2.79	0.62	0.85
N	5	5	5	5	5
Student’s *t*-test (2.306)	1.797	1.068	1.84	2.100	
*F* value (6.39)	2.27	4.68	3.28	1.37	
**Caffeine** ^**[c]**^					
Mean	98.53	98.51	97.80	100.49	99.58
S.D.	2.47	2.45	1.4	0.98	1.22
Variance	6.10	4.9	2.13	0.96	1.49
N	5	5	5	5	5
Student’s *t*-test (2.306)	0.850	0.946	2.091	1.299	
*F* value (6.39)	4.09	3.28	1.43	1.56	
**Chlorpheniramine maleate** ^**[b]**^					
Mean	99.59	100.09	99.80	100.63	99.25
S.D.	0.75	1.02	2.23	1.26	1.28
Variance	0.56	2.04	4.96	1.59	1.65
N	5	5	5	5	5
Student’s *t*-test (2.306)	0.512	0.978	0.522	1.715	
*F* value (6.39)	2.95	1.23	3.00	1.04	

^[a]^ British pharmacopeial method: Titrimetric method with 1 M cerium sulfate until a greenish-yellow color is obtained.

^[b]^ USP pharmacopeial method: Non-aqueous titration using standard 0.1 M perchloric acid using Crystal violet as an indicator.

^[c]^ USP pharmacopeial method: HPLC method using C_8_ column with a mobile phase composed of sodium acetate solution: acetonitrile: tetrahydrofuran (191:5:4, by volume) at a flow rate of 1.0 mL/min and UV detection wavelength at 275.0 nm.

## Conclusion

The continuous development in chemometrics enables the separation and analysis of chemical data beyond univariate analysis. Chemometrics is a potentially successful substitute for expensive chromatographic techniques. The proposed chemometric models have been proficient in swiftly, simply, and consistently measuring PARA, CPM, CAF, and ASC simultaneously with excellent sensitivity and reliability. The greenness of the developed models was considered during their early development stages. Subsequently, they underwent evaluation through the AGREE assessment and penalty point scoring system. Statistically, no significant differences were found between the established and official ones in terms of accuracy, and precision. Thus, the proposed green multivariate models serve as a practical and environmentally conscious option for the standard analysis of PARA, CPM, CAF, and ASC in bulk powder and pharmaceutical formulations.

### Electronic supplementary material

Below is the link to the electronic supplementary material.

Supplementary Material 1

## Data Availability

Data is provided within the manuscript and supplementary materials files.

## Declarations

**Ethics approval and consent to participate**.
