# Supplementary material for: Application of smart chemometric models for spectra resolution and determination of challenging multi-action quaternary mixture: statistical comparison with greenness assessment
Source: BMC Chem. 2024 Mar 2;18(1):44. doi: 10.1186/s13065-024-01148-9 (PMC10909257; doi:10.1186/s13065-024-01148-9)

**Application of Smart Chemometric Models for Spectra Resolution and Determination of Challenging Multi-action Quaternary Mixture: Statistical Comparison with Greenness Assessment**

**Aya A. Mouhamed ^a^, Ahmed H. Nadim ^a*^, Nadia M. Mostafa ^a^, Basma M. Eltanany ^a^**

^a^ Department of Pharmaceutical Analytical Chemistry, Faculty of Pharmacy, Cairo University, Cairo 11562, Egypt.

***Correspondence:**
Ahmed H. Nadim,

Faculty of Pharmacy - Cairo University

Kasr El-Aini St., Cairo 11562, Egypt

ahmed.nagib@pharma.cu.edu.eg

**Supplementary materials**

**Supplementary figures**

**
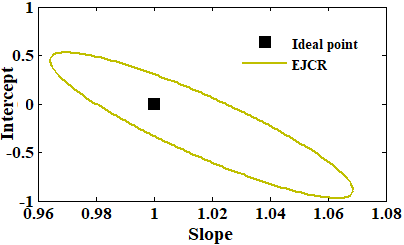
**

**Figure S1.** Elliptical joint confidence region (EJCR) for ANN and MCR-ALS models, same confidence region in the slope-intercept plane, showing that there was no statistical difference between ANN and MCR-ALS models.

**Supplementary tables**

| **Sample No.** | **Nominal concentration (µg mL^-1^)** | | | | **PCR** | | | | **PLS** | | | |
| --- | --- | --- | --- | --- | --- | --- | --- | --- | --- | --- | --- | --- |
|  |  |  |  |  | **Predicted concentration** | | | | **Predicted concentration** | | | |
|  | **PARA** | **ASC** | **CAF** | **CPM** | **PARA** | **ASC** | **CAF** | **CPM** | **PARA** | **ASC** | **CAF** | **CPM** |
| 26 | 20.00 | 15.00 | 2.50 | 1.00 | 19.77 | 14.84 | 2.55 | 1.01 | 19.77 | 14.53 | 2.54 | 1.01 |
| 27 | 4.00 | 15.00 | 2.50 | 9.00 | 3.80 | 14.81 | 2.47 | 8.85 | 3.83 | 14.75 | 2.47 | 8.85 |
| 28 | 8.00 | 8.00 | 8.00 | 8.00 | 7.99 | 7.90 | 7.96 | 8.04 | 8.00 | 7.93 | 7.96 | 8.04 |
| 29 | 12.00 | 9.00 | 7.50 | 5.00 | 11.92 | 9.16 | 7.21 | 4.99 | 11.93 | 9.13 | 7.21 | 4.99 |
| 30 | 20.00 | 3.00 | 12.50 | 1.00 | 11.96 | 3.02 | 12.01 | 1.00 | 19.97 | 3.04 | 12.01 | 1.00 |
| **Sample No.** | **Nominal concentration (µg mL^-1^)** | | | | **MCR-ALS** | | | | **ANN** | | | |
|  |  |  |  |  | **Predicted concentration** | | | | **Predicted concentration** | | | |
|  | **PARA** | **ASC** | **CAF** | **CPM** | **PARA** | **ASC** | **CAF** | **CPM** | **PARA** | **ASC** | **CAF** | **CPM** |
| 26 | 20.00 | 15.00 | 2.50 | 1.00 | 20.17 | 15.05 | 2.47 | 1.02 | 19.96 | 15.00 | 2.50 | 1.00 |
| 27 | 4.00 | 15.00 | 2.50 | 9.00 | 3.96 | 14.60 | 2.41 | 8.69 | 4.00 | 15.00 | 2.50 | 9.00 |
| 28 | 8.00 | 8.00 | 8.00 | 8.00 | 7.85 | 8.15 | 7.84 | 7.95 | 8.00 | 8.00 | 8.00 | 5.12 |
| 29 | 12.00 | 9.00 | 7.50 | 5.00 | 11.97 | 9.04 | 7.21 | 5.00 | 12.13 | 8.96 | 7.65 | 5.00 |
| 30 | 20.00 | 3.00 | 12.50 | 1.00 | 20.09 | 3.00 | 12.43 | 1.01 | 20.00 | 3.00 | 12.5 | 1.00 |

**Table S1. Prediction concentrations of validation set samples using the proposed chemometric models**

**Graphical abstract**


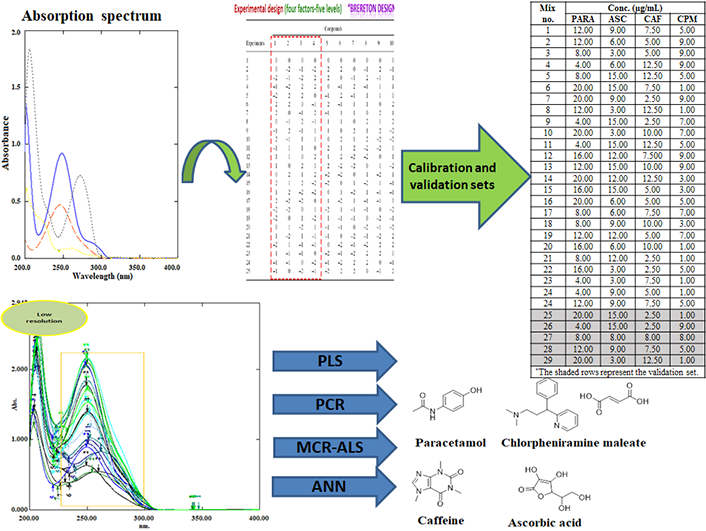

Supplement: Supplementary file 1 — Supplementary Material 1 [file 13065_2024_1148_MOESM1_ESM.docx]
